# Screening of common *CYP1B1* mutations in Iranian POAG patients using a microarray-based PrASE protocol

**Published:** 2008-12-18

**Authors:** Fatemeh Suri, Reza Kalhor, Seyed Jalal Zargar, Navid Nilforooshan, Shahin Yazdani, Hossein Nezari, Seyed Hassan Paylakhi, Mehrnaz Narooie-Nejhad, Behnaz Bayat, Tina Sedaghati, Afshin Ahmadian, Elahe Elahi

**Affiliations:** 1 School of Biology, University College of Science, University of Tehran, Tehran, Iran; 2 Department of Biotechnology, University of Tehran, Tehran, Iran; 3 Department of Ophthalmology, Iran University of Medical Sciences, Hazrat Rasool Hospital, Tehran, Iran; 4 Ophthalmic Research Center, Shaheed Beheshti University of Medical Sciences, Tehran, Iran; 5 Department of Genetics, Faculty of Basic Science, Tarbiat Modares University, Tehran, Iran; 6 National Institute of Genetic Engineering and Biotechnology, Tehran, Iran; 7 Matne Cellule Research Laboratory, Tehran, Iran; 8 Department of Biotechnology, Royal Institute of Technology, Stockholm, Sweden; 9 Bioinformatics Center, Institute of Biochemistry and Biophysics, University of Tehran, Tehran, Iran; 10 Center of Excellence in Biomathematics, School of Mathematics, Statistics and Computer Science, University of Tehran, Tehran, Iran

## Abstract

**Purpose:**

The gene coding cytochrome P4501B1 (*CYP1B1*) has been shown to be a major cause of primary congenital glaucoma in the Iranian population. More recently it was shown to also be important in juvenile-onset open angle glaucoma (JOAG). We aimed to further investigate the role of *CYP1B1* in a larger cohort of primary open angle glaucoma (POAG) patients which included late-onset patients. We also aimed to set up a microarray based protocol for mutation screening with an intent of using the protocol in a future population level screening program.

**Methods:**

Sixty three POAG patients, nine affected family members, and thirty three previously genotyped primary congenital glaucoma (PCG) patients were included in the study. Clinical examination included slit lamp biomicroscopy, IOP measurement, gonioscopic evaluation, fundus examination, and measurement of perimetry. G61E, R368H, R390H, and R469W were screened by a protocol that included multiplexed allele specific amplification in the presence of a protease (PrASE), use of sequence tagged primers, and hybridization to generic arrays on microarray slides. The entire coding sequences of *CYP1B1* and myocilin (*MYOC*) genes were sequenced in all individuals assessed by the microarray assay to carry a mutation. Intragenic single nucleotide polymorphism (SNP) haplotpes were determined for mutated alleles.

**Results:**

Genotypes assessed by the array-based PrASE methodology were in 100% concordance with sequencing results. Seven mutation carrying POAG patients (11.1%) were identified, and their distribution was quite skewed between the juvenile-onset individuals (5/21) as compared to late-onset cases (2/42). Four of the seven mutation carrying Iranian patients harbored two mutated alleles. *CYP1B1* mutated alleles in Iranian PCG and POAG patients shared common haplotypes. *MYOC* mutations were not observed in any of the patients.

**Conclusions:**

The PrASE approach allowed reliable simultaneous genotyping of many individuals. It can be an appropriate tool for screening common mutations in large sample sizes. The results suggest that *CYP1B1* is implicated in POAG among Iranians, notably in the juvenile-onset form. Contrary to POAG patients studied in other populations, many mutation harboring Iranian patients carry two mutated alleles. We propose an explanation for this observation.

## Introduction

Glaucoma is a heterogeneous group of optic neuropathies which manifest by optic nerve head cupping or degeneration of the optic nerve, resulting in a specific pattern of visual field loss [1-3]. The disease affects approximately 65 million people worldwide and is considered the second leading cause of blindness [4]. It is sub-grouped into three major classes on the basis of etiology, anatomy of the anterior chamber, and age of onset [2]. Primary open angle glaucoma (POAG; OMIM 137760) accounts for 70% of glaucoma cases in Caucasian populations and usually affects individuals past the age of 40 [5]. In this form of glaucoma, the anterior chamber angle and the trabecular meshwork appear normal. POAG is sometimes divided into the two sub-classes of adult-onset (or late-onset) and juvenile-onset (JOAG), the latter appearing between early childhood and the age of 40 [6]. Primary congenital glaucoma (PCG; OMIM 231300) is characterized by an anatomic defect of the trabecualr meshwork (trabeculodysgenesis) and an age of onset in the neonatal or infantile period.

Glaucoma in some families demonstrates Mendelian inheritance. Three POAG causing genes have been reported on the basis of genetic studies on multi-case families. The three genes code for myocilin (*MYOC*), optineurin (*OPTN*), and WD repeat containing protein 36 (*WDR36*) [6-8]. Mutations in *MYOC* were the cause of disease in less than 5 percent of POAG patients in various studies [8-10]. Mutations in *OPTN* and *WDR36* have only rarely been found in POAG patients [7,8]. *CYP1B1* (OMIM 601771), encoding cytochrome P4501B1, is a gene commonly associated with primary congenital glaucoma. Mutations in *CYP1B1* account for the disease status of 20 to 100% of PCG patients in various populations [11]. Recently, mutations in *CYP1B1* have been reported in POAG patients [12-16].

The genetic basis of PCG among Iranian patients has been studied and it was found that nearly 70% of Iranian PCG patients carry disease associated mutations in *CYP1B1* [17]. Although nineteen mutations were observed among the 104 patients studied, four (G61E, R368H, R390H, and R469W) constituted 76.2% of the mutated *CYP1B1* alleles identified. More recently [13], it was observed that mutations in *CYP1B1* accounted for disease status in four probands (17.4%) of a cohort of 23 unrelated Iranians affected with JOAG. The mutations observed in the JOAG patients were among the aforementioned common mutations. The relatively large contribution of *CYP1B1* to glaucoma among Iranians suggested by the results of these studies prompted us to further investigate its role in a larger cohort of POAG patients which included late-onset patients. We chose to address only the common mutations with an intent of setting up a neonatal or population level screening protocol to be used in the future. The patients were screened for the mutations by a protocol which included a protease-mediated allele-specific primer extension (PrASE) reaction and hybridization to generic oligonucleotide tag sequences arrayed onto microarray chips [18]. Whereas various technologies have been developed to allow scoring of thousands of sequence variations, the PrASE/microarray protocol is designed for relatively small scale projects wherein far fewer variations are queried in many samples [19]. The results of our effort are presented.

## Methods

This research was performed in accordance with the Helsinki Declaration and with the approval of the ethics board of the International Institute of Genetic Engineering and Biotechnology in Iran. The participants or authorized family members all consented to participate after being informed of the nature of the research. Sixty three unrelated POAG patients were recruited from the ophthalmology divisions of the Hazrat Rasool Hospital (associated with Iran University of Medical Sciences) and the Labbafinejad Hospital (associated with Shaheed Beheshti University of Medical Sciences) in Tehran. The hospitals are national reference centers, and patients from throughout Iran are referred to them. Nine additional affected individuals who were related to the patients were also included in the study. All patients were diagnosed to be affected by at least one of the authors (N.N. or S.Y.) who are glaucoma specialists. Clinical examination and criteria for diagnosis were as previously described [13]. None of the patients had phenotypic characteristics suggestive of PCG such as megalocornea, Descemet's membrane rupture (Haab's striae), corneal scarring, or edema. The patients were recruited consecutively without regard to familial status of disease. They were subsequently queried on the familial status of disease, and those who reported consanguinity between parents and/or incidence of disease among relatives were designated familial. None of the patients nor their family members were included in our previous studies.

The *CYP1B1* mutations addressed by the microarray-based PrASE protocol were c.182G>A causing p.G61E, c.1103G>A causing p.R368H, c.1169G>A causing p.R390H, and c.1405C>T causing p.R469W (reference sequences: NM_000104.2 and NP_000095.1). Polymerase chain reactions (PCR) and hybridization onto chips were done essentially as already described [18]. Briefly, a multiplex outer PCR reaction was performed on extracted DNA, and one microliter of this reaction was used as template for a multiplex nested PCR. Subsequently, the inner PCR amplicons served as template in a PrASE reaction in which extension of mismatched primers was made unlikely by inclusion of a protease that degrades the DNA polymerase. Two allele specific primers for each of the four mutation sites were present in the PrASE reaction, and each of the eight primers contained at its 5′ end a unique sequence complementary to one of the tag oligonucleotides on a generic tag-array. Cy3 labeled dNTPs were also present which allowed fluorescence detection. The products of the PrASE reaction were hybridized to tag-spotted microarrays. Spotting was done using the VersArray ChipWriter (Bio-Rad, Milan, Italy). Each microarray slide was spotted with 48 arrays of 30 tag oligonucleotides in triplicate (array on array). Prior to hybridization, the arrays on each slide were placed into 48 compartments with a home-made silicon rubber mask, and each compartment was used to assess the genotypes of one individual at the four mutation sites. Eight of the tag oligonucleotides were devoted to the present analysis. Fluorescence binding to the spots was detected using VersArray ChipReader (Bio-Rad). Fluorescence signals were measured with the GenePix 5.0 (Medical Devices, Sunnyvale, CA) image analysis software and the allelic fraction (AF) for each individual at each mutation site was calculated. AF is defined as intensity from the normal allele divided by sum of intensities from both alleles. A cluster diagram for each site was created by plotting the AFs of all individuals at that site against the logarithm of the sum of both his signals at that site. If AF values of multiple individuals are well clustered into groups with values approaching zero, one, and an intermediate value, genotypes of individuals in those clusters can be designated, respectively, homozygous for mutant allele, homozygous for normal allele, and heterozygous. The genotype of each individual at each position was called on the basis of his position in the respective cluster diagram. The sequences of all primers used in the PCR and PrASE reactions are presented in Table 1.

**Table 1:** Primer sequences.

**Name**	**Sequence**	**Product size**
**Multiplex outer PCR primers**		
CYP1B1-G61E-out F	5′-CGAGCGAACGAGAGGTGAG-3′	141 bp
CYP1B1-G61E-out R	5′-GCTGGCCACTGTGCATGTG-3′	
CYP1B1-R368H & R390H-out F	5′-GTGCAGGCAGAATTGGATCAG-3′	155 bp
CYP1B1-R368H & R390H-out R	5′-GTGTTGGCAGTGGTGGCATG-3′	
CYP1B1-R469W-out F	5′-GATCCAGCTCGATTCTTGGAC-3′	148 bp
CYP1B1-R469W-out R	5′-GGTGAGCCAGGATGGAGATG-3′	
Multiplex inner PCR primers*		
CYP1B1-G61E-In F	5′-*CACTGCTTCGCAGGCTGACGTTACT*GGCCCACCGCCGCCGCGT-3′	73 bp
CYP1B1-G61E-In R	5′-CGGGCCCGTTTGCGTGGCC-3′	
CYP1B1-R368H-In F	5′-*CACTGCTTCGCAGGCTGACGTTACT*ATCAGGTCGTGGGGAGGGAC-3′	76 bp
CYP1B1-R368H-In R	5′-GGTTGGGCTGGTCACCCATAC-3′	
CYP1B1-R390H-In F	5′-*CACTGCTTCGCAGGCTGACGTTACT*CTGGCCTTCCTTTATGAAGCCA-3′	83 bp
CYP1B1-R390H-In R	5′-GAGGAATAGTGACAGGCACAAAG-3′	
CYP1B1-R469W-In F	5′-*CACTGCTTCGCAGGCTGACGTTACT*AGTGATGATTTTTTCAGTGGGCAA-3′	86 bp
CYP1B1-R469W-In R	5′-CTGCATCTTAGAAAGTTCTTCGC-3′	
Biotinylatad inner primer*	5′- *CACTGCTTCGCAGGCTGACGTTACT*-3′	
**PrASE primers¶**		
CYP1B1-G61E-T22-G	5′-TTACCTATGATTGATCGTGGTGATATCCGTTTGCGTGGCCACTGATCGG-3′	
CYP1B1-G61E-T23-A	5′-GCTGTGGCATTGCAGCAGATTAAGGTTTGCGTGGCCACTGATCGA-3′	
CYP1B1-R368H-T24-G	5′-TGACGTCATTGTAGGCGGAGAGCTAGTCACCCATACAAGGCAGAC-3′	
CYP1B1-R368H-T25-A	5′-TCAATAATCAACGTAAGGCGTTCCTGTCACCCATACAAGGCAGAT-3′	
CYP1B1-R390H-T26-G	5′-TTATCGGCTACATCGGTACTGACTCAGGCACAAAGCTGGAGAAGC-3′	
CYP1B1-R390H-T27-A	5′-CCATTATCGCCTGGTTCATTCGTGAAGGCACAAAGCTGGAGAAGT-3′	
CYP1B1-R469W-T28-C	5′-GGCGTACCTTCGCGGCAGATATAATAGTTCTTCGCCAATGCACCG-3′	
CYP1B1-R469W-T29-T	5′-AACTGAGCCGTAGCCACTGTCTGTCCAGTTCTTCGCCAATGCACCA-3′	

* Common sequence at 5´-termini of inner PCR forward primers and the biotinylated form of the same sequence are italicized. The biotinylated primer was included in the inner PCR reaction to allow capture of PCR products onto strepavidin coated beads before the PrASE reaction. ¶  5´-termini sequences of PrASE primers complementary to the tag sequences on the arrays are underlined.

Coding exons 2 and 3 of *CYP1B1* of all individuals called heterozygous or homozygous at any of the four mutation positions were sequenced by the di-deoxy terminator protocol as previously reported [13]. These exons were also sequenced in four randomly chosen patients without mutations. Sequencing was done to confirm the genotype calls, identify possible second mutations in the gene, and to ascertain intragenic SNP haplotypes associated with the mutant alleles [17]. Additionally, the three exons of *MYOC* were sequenced in all individuals carrying one or two *CYP1B1* mutated alleles as already described [13]. This was done to identify patients who may have carried mutations in both genes [14,20,21]. Intragenic *CYP1B1* and *MYOC* SNP haplotypes of patients carrying *CYP1B1* mutated alleles were ascertained using the PHASE 2.0 software [22,23].

## Results

**Figure 1:**
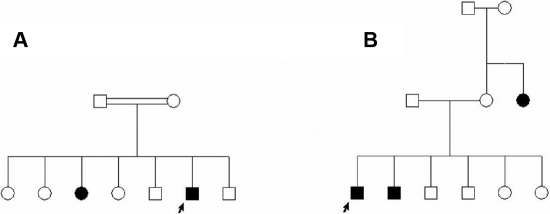
Familial POAG pedigrees. Probands are indicated with arrows. **A**: Pedigree JG-M-4; the age at onset of the two affected siblings was 24 and 25 years. Both carried homozygous *CYP1B1* mutations. **B**: Pedigree LG-R-213; the age at onset of the three affected individuals was 60–68 years. *CYP1B1* mutations were not observed in the proband of this pedigree.

The clinical features of the patients are presented in Table 2. Average age at diagnosis of the patients was 50.3 years old. Age at diagnosis of 21 of the 63 pateints (33.3%) ranged between 6 years and 38 years (average 25.5 years), and these patients were classified as juvenile-onset POAG cases. The average age at diagnosis of the late-onset cases was 62.7 years old. As the patients were recruited without regard to age, a notable fraction, possibly one third, of Iranian POAG patients may have juvenile onset. The heredity status of glaucoma of six of the patients was unknown. Of the remaining 57 patients, 39 (68%) were familial and 18 (32%) were sporadic. The fraction of juvenile and late onset POAG patients who were familial was approximately equal. Two pedigrees in which inheritance was familial are presented in Figure 1.

**Table 2:** Phenotypic features of POAG patients and genotypes of patients with identified mutations.

**Patient**	**Gender**	**Age at diagnosis**	**Affected eye**	**IOP max (mm Hg) right/left**	**C/D ratio right/left**	**Juvenile/ late-onset**	**Inheritance (f/s)**	***CYP1B1* genotype**		***CYP1B1* haplotype¥**	***MYOC* haplotype‡**
								**By PrASE/** **microarray**	**By** **sequencing**		
JG-R-211	M	27	Bilateral	29/31	0.8/0.9	J	f	R390H/R390H	R390H/R390H	H2	H2
JG-L-221	F	37	Bilateral	28/34	0.85/0.95	J	f	G61E/G61E	G61E/G61E	H1	H2
JG-M-4	F	25	Bilateral	32/30	0.9/0.9	J	f	G61E/G61E	G61E/G61E	H1	H1/H2
LG-L-214	M	48	Bilateral	24/28	0.7/0.8	L	f	G61E/+	G61E/+	H1/H3	H2
JG-R-226	M	32	Bilateral	28/28	0.9/0.9	J	f	R368H/+	R368H/E229K	H1/H3	H1/H2
JG-B-5	M	17	Bilateral	22/29	0.3/0.4	J	?	R368H/+	R368H/+	H1	H1
LG-R-234	F	78	left	/34	/0.8	L	s	R368H/+	R368H/+	H1/H2	H2
Patients without identified mutation	33M/ 23F	51.9 (24.9 for JG/ 62.7 for LG)*	39 bilateral /11 right/ 6 left	29.1*	0.8/0.7*	16J/40L	39f/18s/6?				

All data pertain to probands. The asterisk denotes average figures for patients without identifeid mutations. The sharp (hash mark) indicates haplotpes defined by nucleotides at SNP positions IVS1-13, c.142, c.355, c.1294, c.1347 and c.1358 (NM_000104.2); H1: -CCGGTA-; H2: -TGTCCA-; H3: -CCGCCA-. The double dagger (‡) indicates haplotypes defined by nucleotides at SNP positions c.-83, c.227 and IVS2+35 (NM_000261); H1: -GGG-; H2: -GGA-. In the table M=male; F=female; J=juvenile-onset POAG; L=late-onset POAG; f=familial; s=sporadic.

**Figure 2:**
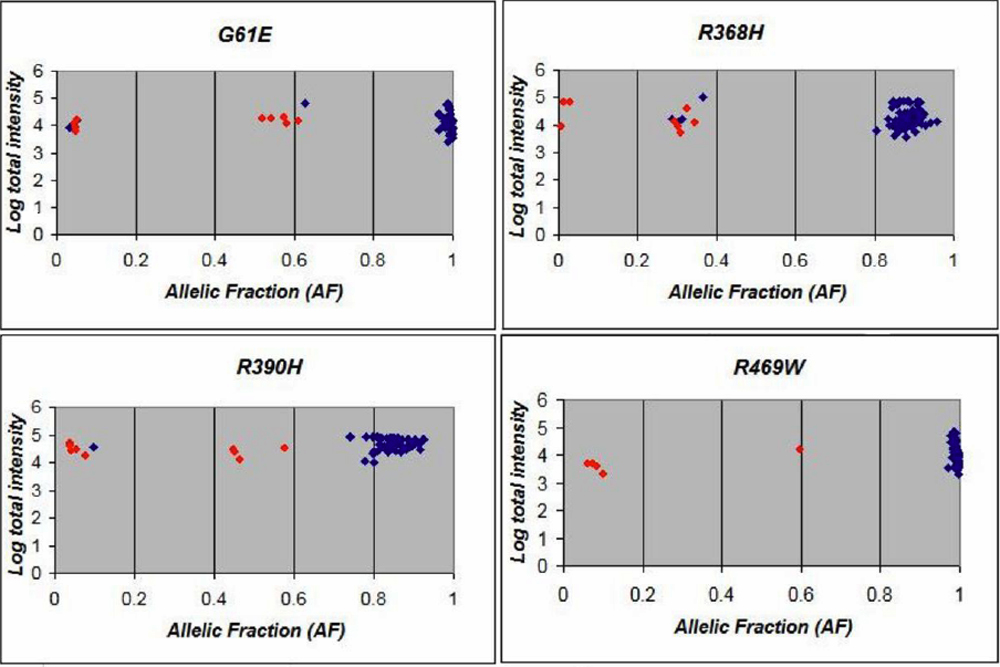
Results of microarray-based PrASE genotypings. Cluster diagrams of genotyping results of 105 individuals for four common *CYP1B1* mutations are shown. Red squares: PCG patients whose genotypes were known because of previous direct sequencing. Blue squares: POAG patients whose genotypes were determined by the PrASE/hybridization reactions.

In addition to the sixty three patients and nine affected relatives, thirty three PCG patients known by sequencing from our previous studies to carry the mutations in the heterozygous or homozygous states were included in the PrASE/microarray analysis to enable formation of stronger clusters [17]. Therefore, 420 genotypes (genotypes of 105 individuals at 4 variant nucleotide sites) were queried by the protocol. The clusters for the four mutations are presented in Figure 2. The three possible genotypes of all the mutations are well clustered, suggesting that genotype calls can be made with confidence. The genotypes of the individuals as ascertained in the clusters could in all cases have been called by visual inspection of the fluorescence intensities on the arrays. Hybridizations on replicate spots were almost identical. All heterozygous and homozygous mutant genotype calls, and absence of the mutations in four individuals were confirmed by sequencing.

We identified mutations in three of the four screened positions. These mutations were found in seven (11.1%) of the 63 patients screened (Table 2). R469W was not observed in any of the patients. One heterozygous carrier of R368H (JG-R-226) was observed to also carry an E229K mutated allele upon sequencing. This mutation has previously been reported to be associated with PCG and POAG [12,15-17,24]. No additional disease associated mutation was observed in the remaining patients by sequencing. Both alleles were mutated in four of the seven patients harboring *CYP1B1* mutations, and only one allele was mutated in the remaining three. G61E, the most frequent mutated *CYP1B1* allele among Iranian PCG patients, was also the most frequently observed mutation among the POAG patients. The JOAG affected sibling of two of the JOAG patients (JG-R-226 and JG-M-4) had the same genotype as the respective proband. The haplotypes associated with most mutations observed in the POAG patients, as defined by six intragenic single nucleotide polymorphisms (SNPs), were the same as previously found to be associated with the same mutations in PCG patients of Iran and other populations (Table 2) [17]. The only exception was the E229K allele identified by sequencing. Multiple origins for this mutation in human history have been suggested based on haplotype data [17,20]. A disease associated *MYOC* mutation was not observed in any of the patients carrying *CYP1B1* mutations. *MYOC* haplotypes in the patients defined by three common intragenic SNPs were the same as previously observed among Iranians (Table 2) [13].

## Discussion

Although the microarray-based PrASE protocol has been previously introduced as a technology alternative to direct sequencing, this work represents its first implementation designed to address a biological question [18,19,25]. The genotypes called were reproducible and in 100% concordance with sequencing data. We believe it is an appropriate tool for screening common mutations in large sample sizes, for example to obtain epidemiological data on carrier frequencies or prevalence rates. Our protocol can easily be upgraded to simultaneously screen a larger number of mutations. Multiplexing the PrASE reaction for up to 75 SNPs has been reported [25,26]. The use of microarray slides spotted with generic tag oligonucleotides allows the flexibility of using the same slides for querying mutations in different genes.

We surmise the *CYP1B1* mutations common in Iranian PCG patients may be associated with disease status in 11.1% of the Iranian POAG patients in our cohort. Five of the patients who carried disease associated *CYP1B1* mutations were among the 21 juvenile-onset (JOAG) subgroup, a proportion (23.8%) similar to the proportion we recently reported for an independent cohort of Iranian JOAG patients (17.4%) [13]. Two of the patients who carried disease associated *CYP1B1* mutations were among the 42 of the late-onset group (4.8%). The difference in proportion of JOAG patients (9/44; including patients of reference 14) and late onset POAG patients (2/42) is statistically significant (p=0.03). A similar trend on the role of *CYP1B1* in JOAG and late onset POAG is revealed upon analysis of data presented in two previous studies which included both juvenile and late-onset cases [12,16]. These observations suggest that in addition to the significant role that *CYP1B1* has in PCG, it may also have roles in juvenile and late-onset POAG and that its role in JOAG may be more notable than its role in late-onset POAG. The haplotype analysis presented here show that identical mutated *CYP1B1* alleles may be involved in the etiology of the various forms of glaucoma, suggesting that other genetic and/or environmental factors can affect the course of disease development.

In three studies on the role of *CYP1B1* in POAG of patients from various populations excluding the studies on Iranian patients, 29 of 518 patients (5.6%) were reported to carry mutations in the gene [12,15,16]. Among the 29 patients, only three carried two mutated alleles. The results at first appearance seem to be in contrast to the findings on the Iranian population wherein eight of the eleven POAG patients with *CYP1B1* mutations carried two mutated alleles [13 and present study]. However, it is possible that this discordance is due to the combined effects of difference in role of *CYP1B1* mutations in juvenile-onset and late-onset POAG, and difference in proportions of these classes of patients in the populations. In fact, The Genetics Home Reference site, sponsored by the US National Library of Medicine, states that juvenile open angle glaucoma affects about 1 in 50,000 people, whereas open angle glaucoma affects about one percent of individuals past the age of 40 years. Our patient recruitment suggests that a notably higher proportion, possibly one third, of Iranian POAG cases may be juvenile-onset cases (see results). We suggest that harboring two mutated *CYP1B1* alleles will most likely result in the PCG phenotype. If genetic background and/or environmental factors preclude this outcome, the POAG phenotype may arise, most likely the juvenile form. All eight Iranian POAG patients carrying two mutated alleles were juvenile-onset cases, whereas only one patient with a single mutated allele was juvenile onset. Similarly, age at diagnosis of two of the three patients in the other populations who carried two mutated alleles was 13 and 18 years [12,16]. Individuals carrying one mutated *CYP1B1* allele may be asymptomatic or only mildly symptomatic. If they develop glaucoma, it is more likely that they develop late-onset POAG rather than the more severe congenital or even juvenile forms of glaucoma.

In Western populations wherein consanguineous marriages are rare and in populations wherein *CYP1B1* is a cause of PCG in a relatively low percent of patients, possibly indicating low frequency of *CYP1B1* mutated alleles in those populations, individuals who carry two mutated alleles will be correspondingly few and most of these will develop PCG. It will be more likely to identify POAG patients with one rather than two mutated alleles in these populations. However, in a population like Iran wherein extensive inbreeding exists and wherein mutated *CYP1B1* alleles may be frequent, there are expected to be a larger number of patients with two mutated *CYP1B1* alleles who do not develop PCG and proceed to develop POAG at a later age. The heteorozygotes are likely to have milder symptoms and remain undiagnosed. These considerations may explain why autosomal recessive inheritance for JOAG and JOAG/PCG mixed pedigrees are often observed in the Iranian population [13]. They may also shed light on the pathogenic effects of *CYP1B1* mutations. It will be of interest to learn the *CYP1B1* genotypes and inheritance pattern of JOAG in other populations with high rates of consanguineous marriages and high frequencies of mutated *CYP1B1* alleles.

We conclude that *CYP1B1* is important in the etiology of POAG, particularly the juvenile form, in the Iranian population. Incomplete penetrance for *CYP1B1* mutations with regard to PCG has been well documented [27]. However the results of the present study suggest that long-term follow up of unaffected individuals with an affected genotype may result in diagnosis of juvenile or late onset POAG. As all forms of glaucoma are devastating diseases which may lead to blindness and have notable social and economic consequences, it is recommended that the gene be screened for mutations in individuals at risk, and that phenotypically affected and unaffected individuals carrying mutated alleles be kept under medical surveillance.
